# NLRP3/Caspase-1 inflammasome activation is decreased in alveolar macrophages in patients with lung cancer

**DOI:** 10.1371/journal.pone.0205242

**Published:** 2018-10-26

**Authors:** Ismini Lasithiotaki, Eliza Tsitoura, Katerina D. Samara, Athina Trachalaki, Irini Charalambous, Nikolaos Tzanakis, Katerina M. Antoniou

**Affiliations:** 1 Laboratory of Cellular and Molecular Pulmonology, Medical School, University of Crete, Heraklion, Crete, Greece; 2 Department of Thoracic Medicine, Interstitial Lung Disease Unit, University Hospital of Heraklion, Crete, Greece; University of South Alabama Mitchell Cancer Institute, UNITED STATES

## Abstract

Lung cancer (LC) remains the leading cause of cancer-related mortality. The interaction of cancer cells with their microenvironment, results in tumor escape or elimination. Alveolar macrophages (AMs) play a significant role in lung immunoregulation, however their role in LC has been outshined by the study of tumor associated macrophages. Inflammasomes are key components of innate immune responses and can exert either tumor-suppressive or oncogenic functions, while their role in lung cancer is largely unknown. We thus investigated the NLRP3 pathway in Bronchoalveolar Lavage derived alveolar macrophages and peripheral blood leukocytes from patients with primary lung cancer and healthy individuals. IL-1β and IL-18 secretion was significantly higher in unstimulated peripheral blood leukocytes from LC patients, while IL-1β secretion could be further increased upon NLRP3 stimulation. In contrast, in LC AMs, we observed a different profile of IL-1β secretion, characterized mainly by the impairment of IL-1β production in NLRP3 stimulated cells. AMs also exhibited an impaired TLR4/LPS pathway as shown by the reduced induction of IL-6 and TNF-α. Our results support the hypothesis of tumour induced immunosuppression in the lung microenvironment and may provide novel targets for cancer immunotherapy.

## Introduction

Lung cancer remains the leading cause in cancer-related mortality in both males and females. Approximately 85% of lung tumours are non-small cell lung cancer (NSCLC), including adenocarcinoma, squamous cell carcinoma and large cell carcinoma [1]. Although the majority of lung cancer patients are smokers, only a minority among smokers will develop this disease, strongly suggesting that additional environmental determinants including infections, in a background of genetic susceptibility, drive disease initiation and progression[2].

The crosstalk between inflammation and tumorigenesis is a field of active research and although cell-autonomous aberrations are considered the initiators of cancer, chronic inflammation has been shown to promote cancer initiation and progression [3]. Recent studies in tumor immunosurveilance propose a strong relationship between lung cancer risk factors and alterations in inflammatory cytokine levels, oxidative stress markers and immune cell composition [4]. The interaction of cancer cells with their microenvironment, particularly with immune cells results in stimulatory or inhibitory effects that result either in tumor escape or elimination [5] [6].

Alveolar macrophages (AMs) play a critical role in lung immunoregulation and potentially in the prevention of lung diseases, including lung cancer. AMs regulate local inflammatory reactions via the release of cytokines and phagocytosis. Pro-inflammatory macrophages induce synthesis and upregulation of several pro-inflammatory cytokines and chemokines, through activation of the NF-kB and MAPK cascades. Key among these are TNF-α, IL-12, IL-6, CCL2 and interleukin-1β (IL-1β). At the other extreme, macrophages are alternatively polarized to the anti-inflammatory states by stimuli such as IL-4, IL-13, IL-10 or glucocorticoid hormones[7, 8]. These macrophages upregulate IL-1 receptor antagonist and downregulate IL-1β and other pro-inflammatory cytokines. Studies investigating the role of AMs in lung cancer have provided inconsistent results. Whilst some studies report increased cytotoxic activity and antitumor effects after AMs activation, others have reported decreased cytotoxic activity and pro-tumor effects [9] [10] [11]. A dual role for macrophages in lung cancer has therefore been suggested.

A central mechanism driving inflammation in immune cells is orchestrated by the inflammasome. Inflammasomes are multi-molecular protein complexes responsible for Caspase-1 driven activation of the pro-inflammatory cytokine IL-1β and IL-18[12]. They are involved in the innate immunity by recognizing pathogen-associated molecular patterns (bacteria, viruses and fungi) and intracellular and extracellular damage-associated molecular patterns [12]. IL-1β and IL-18 exert pleiotropic effects in inflammation and tumorigenesis. Mature IL-1β, primarily produced by monocytes and macrophages, albeit absent under normal conditions, shows an important inducible transcription during inflammation and stress [13]. The most well studied Inflammasomes are NLRP3, activated by various stimuli, NLRC4 activated by bacterial flagellin and AIM2 activated by cytosolic double stranded DNA[14].

NLRP3 is activated by the widest array of stimuli, microbial or sterile. In most cells, NLRP3 must be primed by a toll-like receptor (TLR)/nuclear factor-κB signal [15], to upregulate the expression of pro-IL-1β, pro-IL-18 and NLRP3. Once primed a second signal, by various pathogen-associated molecular patterns (PAMPs) and damage-associated molecular patterns (DAMPs), pore-forming toxins, adenosine triphosphate (ATP), and particulate crystals leads to the cleavage of active IL-1β and IL-18, by caspase-1[14].

Inflammasomes can modulate tumor immunity during carcinogenesis playing a role in immunosuppression, and could also formulate the response to anti-tumor vaccines [9, 16]. The effects of NLRP3 activation in neoplastic progression may be contingent on the differential roles played by immunity and inflammation in each specific malignancy[17]. In human lung cancer, the field of inflammasome mediators and inflammasome activation remains relatively unknown. Interestingly, exploratory data from a randomized trial proposed that IL-1β inhibition results in decreased incidence of Lung cancer in patients with high CRP[18].

In this view, we aimed to investigate an IL-1β specific pathway, the NLRP3 inflammasome, in lung cancer peripheral blood leukocytes and alveolar macrophages obtained from bronchoalveolar lavage and to characterize this inflammatory process in primary lung cancer in the periphery and locally in the lungs. Our results showed distinct NLRP3/Caspase 1 dependent IL-1β maturation in alveolar macrophages relative to peripheral blood cells with strong stimulation in the periphery and reduced stimulation locally in the lungs of NSCLC and small cell lung cancer (SCLC) groups.

## Patients and methods

### Patients

Thirty-one (31) consecutive subjects were enrolled in this study in three groups: non small cell lung cancer (NSCLC) group (n = 15), small cell lung cancer (SCLC) group (N = 4) and Control group (n = 12). Lung cancer patients underwent bronchoscopy at the diagnosis of cancer and none received lung cancer therapy at the time of the experiments. The control group consisted of patients undergoing bronchoscopy for the investigation of haemoptysis. During the investigation of haemoptysis no underlying disease was discovered, the bronchoscopy findings and the cytology results were normal. No inhaled medications [19] were used by any of the participants before and at the time of sample collection. Subjects who had experienced respiratory infections 6 weeks prior to bronchoscopy were excluded. Informed consent was obtained from all patients. The study was approved by the Ethics Committee of the University Hospital of Heraklion, Crete, Greece (17517/19-12-2013).

### Biological samples and processing

Bronchoalveolar Lavage Fluid (BALF) was obtained from all patients and controls and isolation of macrophage population was performed as previously described [20, 21]. In brief, a flexible bronchoscope was wedged into a sub segmental bronchus of a predetermined region of interest based on radiographical findings. A BALF technique was performed by instilling a total of 240 mL of normal saline in 60-mL aliquots, each retrieved by low suction. Volume received was the same for all samples. The BALF fractions were pooled and split equally into two samples. One sample was sent to the clinical microbiology and cytology laboratory and the other was kept at room temperature (RT) and used for this research. BALF was passed through a 70nm filter (Millipore) to isolate cells in suspension from debris and mucus. To pellet cells, samples were centrifuged at 1,500 rpm for five minutes at RT. The supernatant was discarded and the cells were resuspended in 4 ml RPMI medium, 10% FBS, 10x penicillin/streptomycin, followed by cell count in a Neubauer haemocytometer. Equal amounts of BALF sample cells were loaded onto six-well plates, using RPMI supplemented with 10% heat-inactivated FBS as culture medium. BALF sample cells were observed using an inverted microscope, to verify adherence of macrophages.

Heparin-anticoagulated whole blood from patients and controls were obtained at room temperature. Whole blood was used after Red Blood Cell lysis (RBC lysis Buffer, eBioscience Product No 00-4333-57). A white blood cell count, in parallel with the BALF cell count, was performed using a Neubauer chamber. Equal amounts of blood and BALF sample cells were loaded onto six well plates, using RPMI complete as culture medium.

### NLRP3 inflammasome activation

For NLRP3 stimulation a protocol already published by our group was used[22]. In brief, TLR4 stimulation with LPS (MERCK, Lipopolysaccharide, *E*. *coli* O111, cat#437627-5MG) (250 pg/ml, 2hrs incubated at a 37°C/5% CO_2_ humidified incubator) was followed by NLRP3-inflammasome activation with a 5mM ATP pulse (Sigma Aldrich, Adenosine 5′-triphosphate disodium salt hydrate cat# A2383) for 20min at 37°C/5% CO_2_, in the presence or absence of 10μΜ a selective, irreversible caspase-1 inhibitor (Calbiochem, Caspase-1 Inhibitor II, CAS 178603-78-6, 5MG, as previously described (S1 Fig) [22, 23]. Cells were scraped and harvested by centrifugation at 1000 rpm at 4°C. Supernatants were collected and cell pellets were lysed in RIPA buffer (Sigma Aldrich cat#R0278), according to the manufacturer’s instructions. Supernatants and cell extracts were stored at -80°C.

### Inflammasome pathway gene expression

Total RNA extraction was performed with Tri Reagent, MBL followed by Nucleospin RNA II kit, Macherey Nagel (Ref number 740955.50). cDNA synthesis was performed with Maxima First Strand cDNA Synthesis Kit, Thermo Scientific (cat. Number K1641). qPCR Master Mix, Thermo Scientific(cat. Number K0222), using the MxPro 3000P, Agilent Technologies for the evaluation of *NLRP3*, *IL-18*, *Caspase-1* expression by qRT2-PCR. *Gapdh* was used as housekeeping gene. Primer sequences used for *NLRP3*: F5’GATCTTCGCTGCGATCAACAG-3’, R5’CGTGCATTATCTGAACCCCAC3’, for *IL-18*: F*5’-GCTTCCTCTCGCAACAAACT-3’*, F*5’- TTGATGCAATTGTCTTCTACTGG-3’*, for *Caspase-1* F5’- CAGAGCTGTGCAGATGAGT-3’, R5’- CTGCAGCCACTGGTTCTGT-3*’* and for *Gapdh* F5’-GGAAGGTGAAGGTCGGAGTCA-3’, R5’-GTCATTGATGGCAACAATATCCACT-3’. All reactions were run in duplicates and transcript levels were calculated and normalized to *Gapdh* as well as the appropriate calibrators, using the Pfaffl method for relative quantification. Normalized values were calculated using the following equation: Fold change = eff goi^(Ct goi calibrator-Ct goi test sample)/eff ref^(Ct ref calibrator-Ct ref test sample).

### Intracellular IL-1β quantification

Intracellular IL-1β protein levels (normalized to beta actin) were assessed by immunoblotting, as previously described [21]. In detail, BALF macrophages and blood leukocytes from 19 lung cancer patients and 12 control subjects were homogenised in RIPA buffer, and 50 μg of protein sample were separated by 12.5% SDS-polyacrylamide gel electrophoresis. Protein were subsequently transferred to nitrocellulose membranes and mature IL-1β protein was detected with rabbit polyclonal antibody against IL-1β (17kDa protein) (Cell Signalling Technology cat#2022) and enhanced chemiluminescence. Mouse anti-actin antibody (MAB 1501, Chemicon, Temecula, CA) was used to normalize IL-1β expression. Films were scanned and the protein lanes were quantified using the Photoshop CS2 image analysis software (Adobe Systems Inc., CA).

### Quantification of secreted cytokine levels

Secreted TNF-α, IL-1β, IL-18 and IL-6 in all supernatants were assessed by ELISA using commercial kits (eBioscience, Human IL-1β ELISA Ready-set-go! cat#88701088, Human IL-6 ELISA Ready-set-go! Cat#88-7066-86, Human TNF-α ELISA Ready-set-go! Cat#88734688, Human IL-18 Platinum ELISA cat#BMS267/2), according to the manufacturer’s instructions. Results were normalized to represent pg/mL per one million (10^6^) cells.

All reagents were pyrogen-free for all studies. No contribution of independent activation of macrophages s by contaminants was observed.

### Statistical analysis

IL-1β and IL-6 secreted, IL-1β cytosolic levels as well as qRT-PCR relative expression results were evaluated using one-sample Kolmogorov-Smirnov goodness of fit test, Student’s t-test and Mann-Whitney U test. Values reported are means ± SD (standard deviation). Statistical analysis was carried out using SPSS 17.0 Chicago IL, USA. Statistical significance was set at the 95% level (P-value < 0.05).

## Results

### Patient characteristics

Demographics of patients and controls are shown in Table 1. Fifteen (15) patients were diagnosed with NSCLC and four (4) with SCLC. Patients with both lung cancer groups compared to controls showed no difference in age, smoking status, sex and BAL fluid cell populations (Table 2). The control group included 4 current smoker females and 7 current smoker males, the NSCLC group included 1 former smoker female and 3 former smoker males as well as 2 current smoker females and 7 current smoker males, the SCLC group included 1 current smoker female and 2 current smoker male. Two lung cancer patients and two control patients had Chronic Obstructive Pulmonary Disorder (COPD) according to GOLD criteria[24], while no other overt pulmonary comorbidities were discovered. The main histological type of NSCLC was adenocarcinoma (11/15 patients) followed by squamous carcinoma (3/15) and large cell carcinoma (1/15). No differences arose in any of the studied cytokines based on histological subtype.

**Table 1:** Clinical characteristics of the studied populations.

Characteristics	Controls	NSCLC	SCLC	p value
Number	12	15	4	
Age *	58 ± 10.6	63.73 ± 12.5	61.3 ± 15.5	NS
Gender ** (male/female)	7/5	14/1	3/1	NS
Smoking status ** (non/ former/current)	1/ 0 /11	1/ 4 /9	1/ 0 /3	NS
Pack years *	30 ± 24	46 ± 28	61 ± 10	NS
COPD (Y/N)	2/10	2/13	0/4	NS

Values are expressed as means ± standard deviations.

*t-test

**χ^2^ test

*P* < 0.05 is considered statistically significant.

**Table 2:** BALF cell population characteristics of all subjects studied.

	Controls	NSCLC	SCLC	p value
N	12	15	4	
Macrophages (%)	75.00±9.35	79.21±8.99	78.82±9.10	NS
Macrophages total cell count	840625± 104797.9	1386835 ± 157399.9	1009553 ± 116555.8	
Neutrophils (%)	6.75±3.50	7.32±1.99	8.10±2.00	NS
Neutrophils total cell count	75656.25± 39229.17	128161 ± 34841.58	103747.5 ± 25616.67	
Lymphocytes (%)	18.25±6.95	14.89±1.88	13.63±3.32	NS
Lymphocytes total cell count	204552.1 ± 77897.92	260699.1 ± 32915.67	174577.6 ± 42523.67	
Eosinophils (%)	0.00±0.00	0.00±0.00	0.00±0.00	-
Eosinophils total cell count	0±0	0±0	0±0	

Values are expressed as means ± standard deviations. NS; not significant.

### IL-1β and IL-18 secretion is activated in PBMCs and alveolar macrophages in NSCLC and SCLC

**Fig 1:**
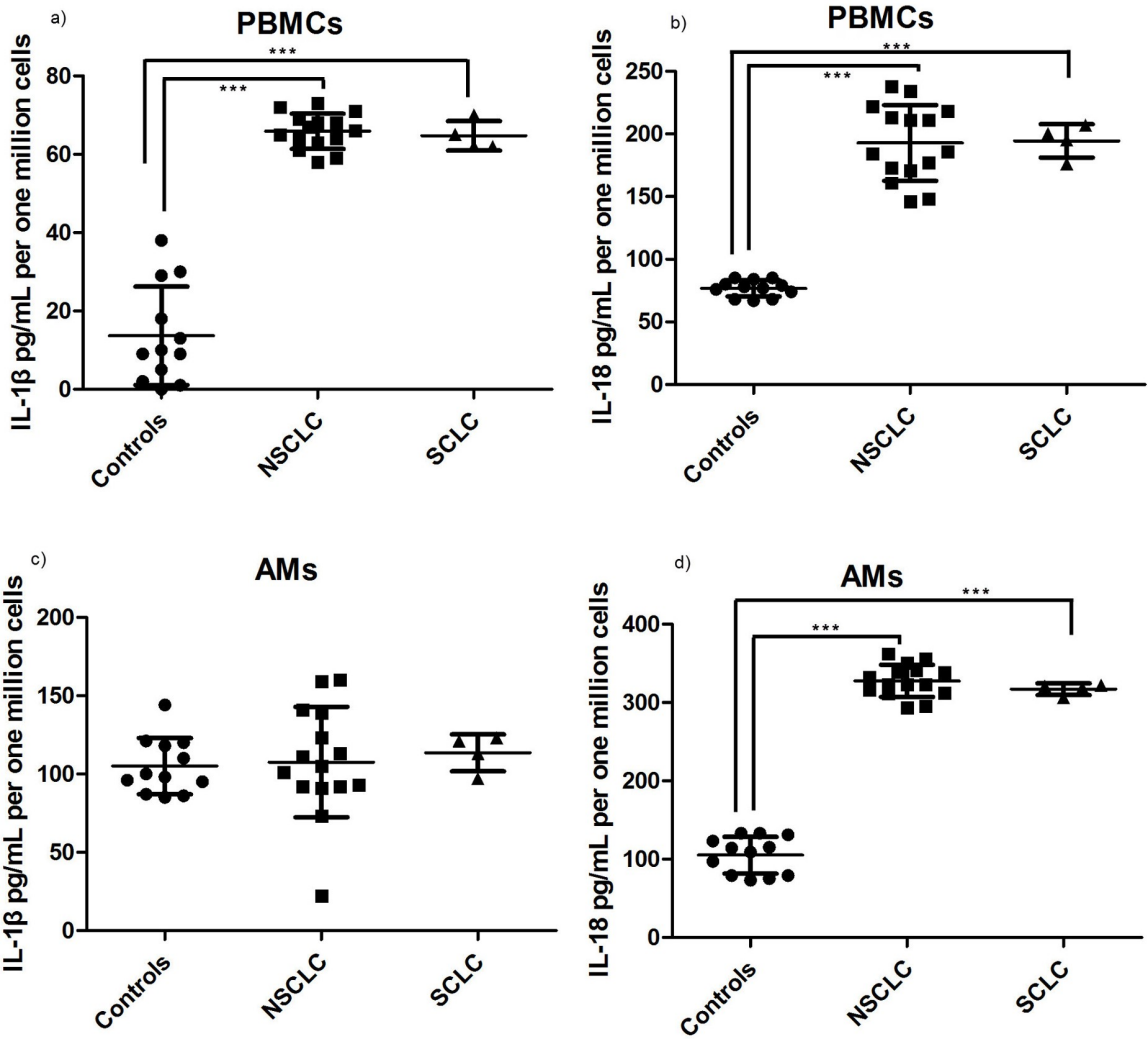
IL-1β and IL-18 secretion by PBMCs and AMs in NSCLC and SCLC relative to controls. Concentration of IL-1β and IL-18 levels measured in the supernatants of unstimulated cultures of PBMCs and AMs following a 2.3h period. a) IL-1β in PBMCs, b) IL-18 in PBMCs, c) IL-1β in AMs and d) IL-18 in AMs. Results are shown as mean+/- SD pg/ml, normalized per million cells cultured.

Initially, we measured the secretion of IL-1β and IL-18 by unstimulated peripheral blood leukocytes (PBMCs) and alveolar macrophages (AMs) as an indirect indication of inflammasome activity in the periphery and locally in the lungs of the patients respectively. PBMCs from NSCLC and SCLC groups released higher levels of IL-1β and IL-18 in the culture medium compared to controls (Fig 1A and 1B). Alveolar macrophages from the NSCLC and SCLC groups also produced higher amounts of IL-18 but not IL-1β (Fig 1C and 1D). NLRP3 requires a two-step process. In the priming step Il-1b and other key components of the inflammasome are overexpressed. Thereafter in order to determine that the first step occurred properly we measured cytosolic levels of IL-1b. Interestingly, only control PBMCs and AMs retained mature IL-1β in their cytoplasm whereas no intracellular levels of mature IL-1β were detectable in the cells from NSCLC and SCLC (S2A and S2B Fig).

### IL-1β and IL-18 secretion via NLRP3 stimulation is decreased in NSCLC and SCLC AMs

Next, we assessed the production of mature IL-1β and IL-18 by PBMCs and AMs following NLRP3 inflammasome stimulation. We used a TLR4 agonist as priming signal, and ATP as the second “danger” signal as described in materials and methods and S1 Fig. To verify that IL-1β and IL-18 secretion was due to LPS/ATP activation of the NLRP3 inflammasome we also stimulated the cells in the presence of Caspase 1 inhibitor I.

**Fig 2:**
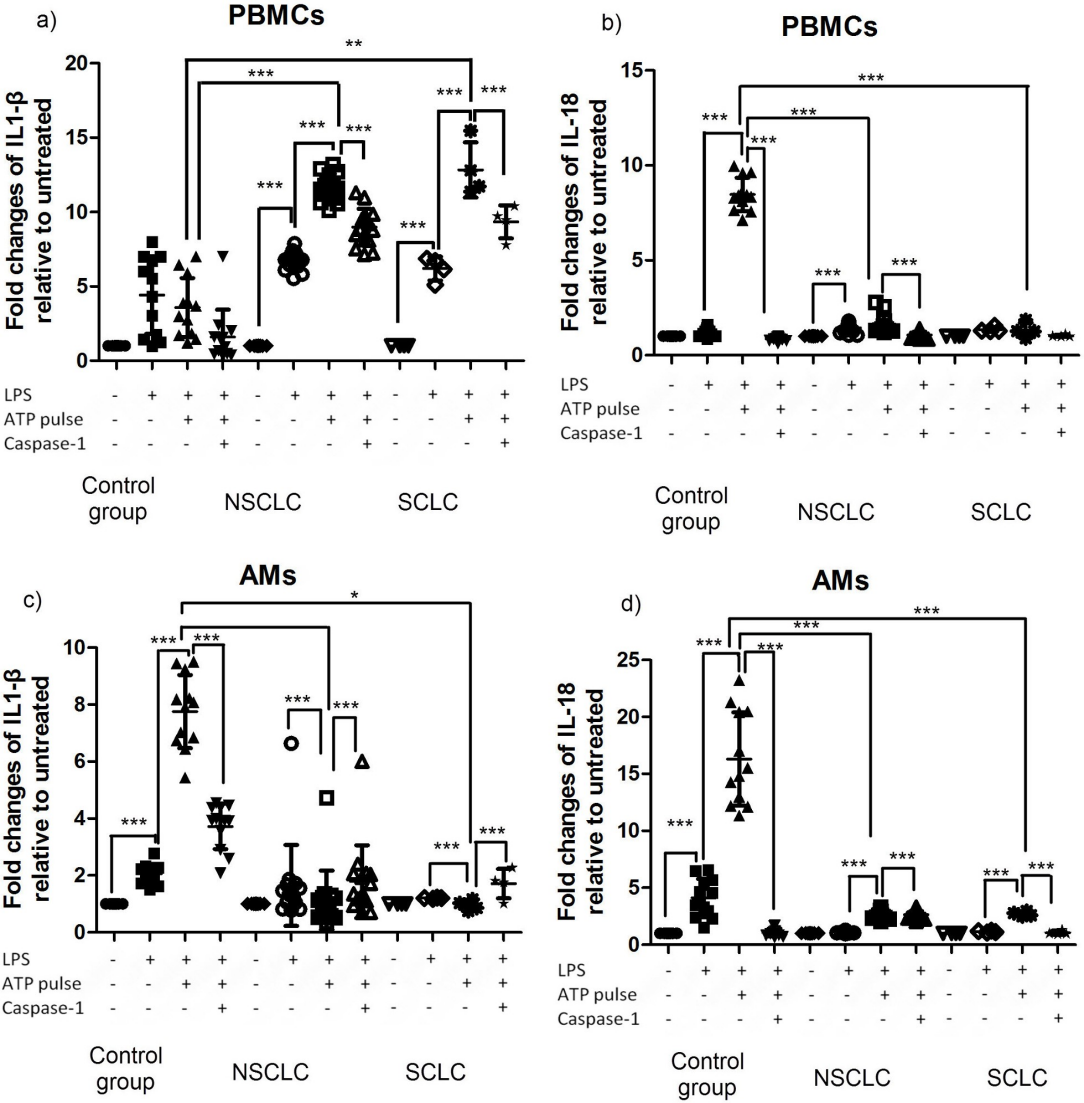
Inflammasome activation of PBMCs and AMs in NSCLC and SCLC relative to controls. Induction of IL-1β and IL-18 secretion by LPS, LPS +ATP and LPS+ATP+ Caspase1 inhibitor I. LPS 250 pg/ml, 2hrs incubated at a 37oC/5% CO2 humidified incubator, followed by a 5mM ATP pulse for 20min at 37oC/5% CO2, in the presence or absence of 10μΜ caspase-1 inhibitor. a) IL-1β in PBMCs, b) IL-18 in PBMCs, c) IL-1β in AMs and d) IL-18 in AMs. Results are shown as mean+/- SD pg/ml, normalized to unstimulated.

We observed that in PBMCs from NSCLC and SCLC, the already elevated IL-1β secretion, could be further stimulated in an NLRP3/Caspase-1 dependent fashion (Fig 2A) in contrast to the levels of IL-18 (Fig 2B). In AMs, we observed a different profile of IL-1β secretion than in PBMCs. IL-1β secretion did not increase upon NLRP3/Caspase-1 inflammasome stimulation in NSCLCs and SCLCs (Fig 2C) similarly to IL-18 (Fig 2D). Overall, controls followed a canonical IL-1β and IL-18 secretion following NLRP3/Casp1 inflammasome profile in AMs.

### mRNA levels of NLRP3 are significantly reduced in AMs in NSCLC and SCLC

**Fig 3:**
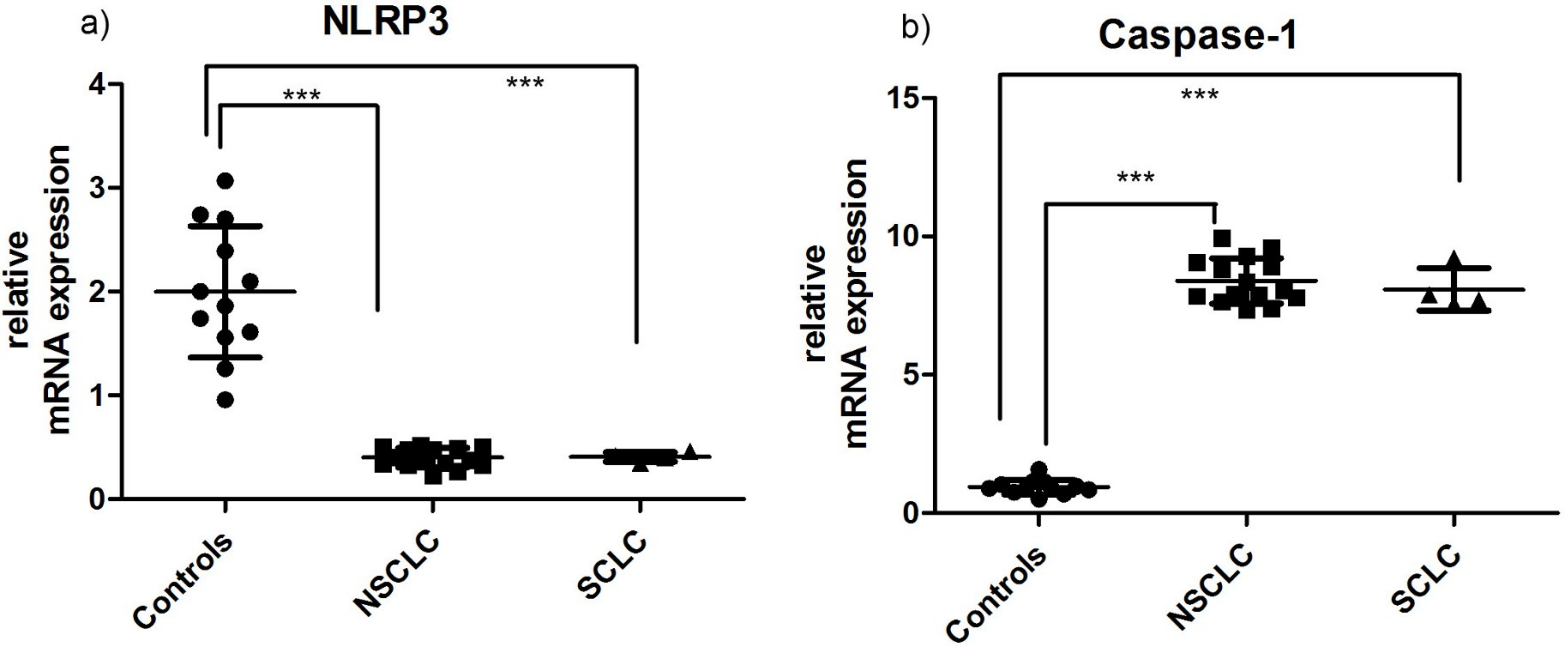
mRNA expression of NLRP3 and Caspase-1 in AMs. Transcript levels of a) NLRP3 and b) Caspase 1 in unstimulated AMs from controls, NSCLC and SCLC.

To further investigate the reduced production of mature IL-1β and IL-18 by AMs we examined the expression of the NLRP3 by RT-PCR. Our results showed that NLRP3 mRNA was significantly downregulated in AMs from NSCLC and SCLC (Fig 3A). Interestingly, Caspase-1 mRNA was significantly upregulated in NSCLC and SCLC (Fig 3B).

### Pro-inflammatory cytokines IL-6 and TNF-α stimulation is also decreased in AMs from NSCLCs and SCLCs

**Fig 4:**
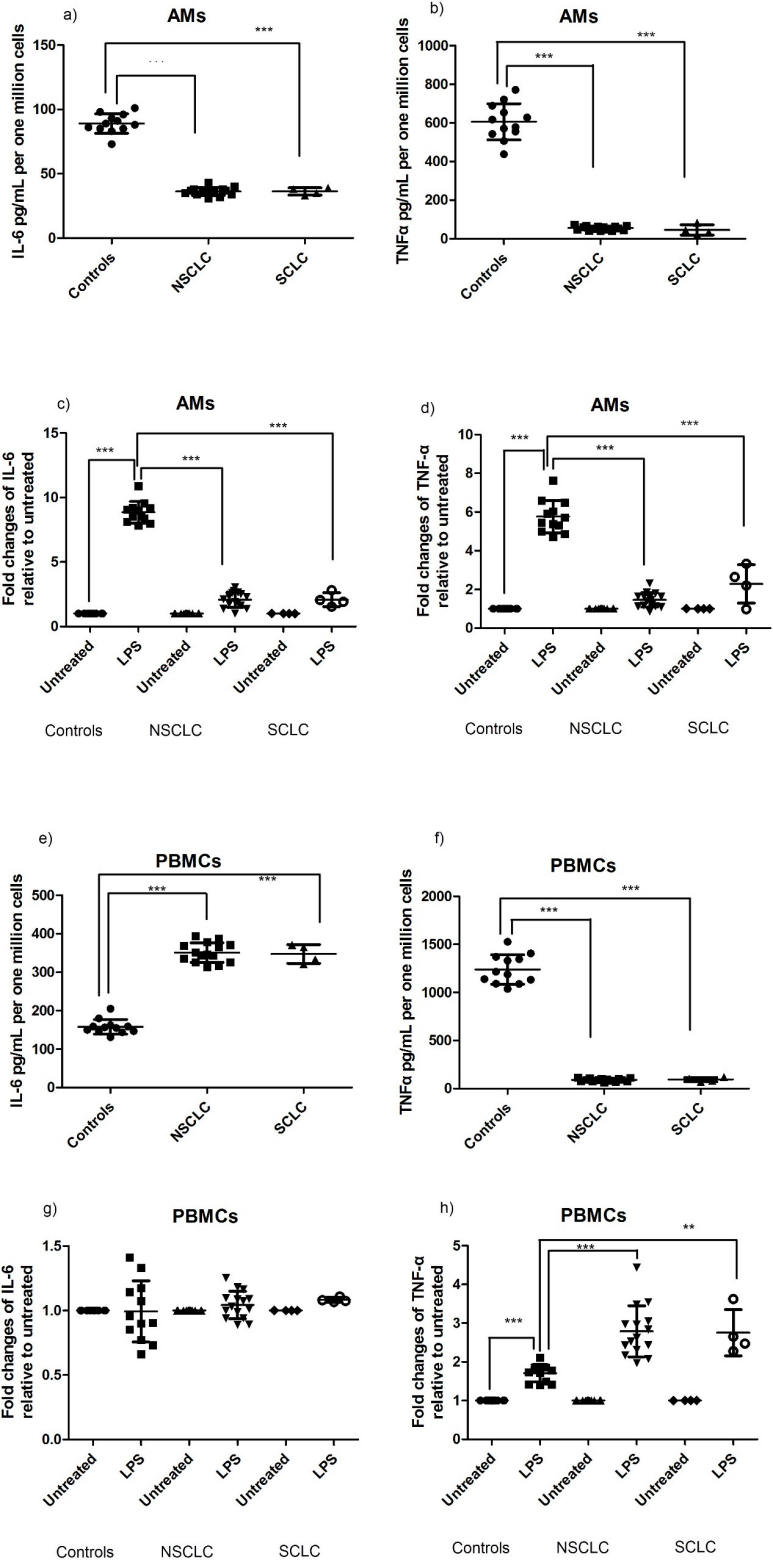
IL-6 and TNF-α secretion by AMs and PBMCs in NSCLC and SCLC relative to controls. Concentration of IL-6 (a) and TNF-α (b) levels measured in the supernatants of unstimulated cultures of AMs following a 2.3h period. Induction of IL-6 (c) and TNF-α (d) in AMs supernatants by LPS—LPS 250 pg/ml, 2hrs incubated at a 37oC/5% CO2 humidified incubator. Concentration of IL-6 (e) and TNF-α (f) levels measured in the supernatants of unstimulated cultures of PBMCs following a 2.3h period. Induction of IL-6 (g) and TNF-α (h) in PBMCs supernatants by LPS- LPS 250 pg/ml, 2hrs incubated at a 37oC/5% CO2 humidified incubator. Results in a, b, e and f are shown as mean+/- SD pg/ml, normalized per million cells cultured. Results in c, d, g and h are shown as mean+/- SD pg/ml, normalized to unstimulated.

Next, we tested the levels of IL-6 and TNF-α in the unstimulated and the LPS stimulated cells. AMs from NSCLCs and SCLCs secreted significantly lower amounts of IL-6 and TNF-α than the controls (Fig 4A and 4B). In addition, LPS stimulation resulted in a significantly lower induction of IL-6 and TNF-α expression by AMs from NSCLCs and SCLCs relative to controls (Fig 4C and 4D).

Unstimulated lung cancer peripheral leukocytes produced higher IL-6 levels than the controls (Fig 4E) while under the experimental conditions used the 2 h stimulation of PBMCs with a low dose of LPS did not induce IL-6 secretion in either the control or the patient groups (Fig 4G). In contrast, TNF-α secretion by the unstimulated PBMCs was significantly lower in the lung cancer patients relative to controls (Fig 4F). TNF-α levels in the PBMCs could be further induced by low dose of LPS in all groups and although stimulation was stronger in both NSCLC and SCLC (Fig 4H), overall TNF-α levels remained very low in the lung cancer groups.

No significant difference between the COPD patients and any other group (control or lung cancer) was found between baseline or after NLPR3 induction in any of the studied cytokines.

## Discussion

The NLRP3 inflammasome expression and role in lung cancer is relatively unknown, while inflammatory reactions can exert a dual influence on tumor growth and progression (14). Recently, studies have proposed a strong relationship between lung cancer risk factors and alterations in inflammatory cytokine levels, oxidative stress markers and immune cell composition [4]. In this view, we aimed to investigate a central innate immune response pathway, the NLRP3 inflammasome, in lung cancer PBMCs and AMs, in order to gain insight on inflammatory processes related to LC. Our primary finding, that alveolar macrophages show decreased transcriptional and secretional TLR4 mediated NLRP3 activation markers, suggests that in human lung cancer innate immune responses in alveolar macrophages are compromised. Additionally, the LC group exhibited a pro-inflammatory state systemically, which could be attributed to the pro-inflammatory state of the patients that lead to the LC, supporting the inflammation induced cancer hypothesis[25].

It has been suggested that chronic inflammation can cause immune suppression in cancers [7], while differential expression of inflammasomes has been observed in various human lung cancer lines and tissues [26]. Variations in inflammasome response have been shown to occur depending on histological subtype, staging and invasive potential [27, 28]. Our results show that locally LC AMs were unable to activate the NLRP3 inflammasome, while at baseline they produced similar amounts of IL-1β and higher amounts of IL-18 compared to control. Additionally, AMs produced low levels of IL-6 and TNF-α which did not increase upon LPS stimulation. Our results suggest that AMs in lung cancer exhibit an altered polarization status[29], similar to tumor associated macrophages (TAMs).

The phenotype of monocytes/macrophages plays a significant role in tumor progression. Depending on the type of the malignancy and the prevalent polarization status, macrophages are associated with favorable or unfavorable clinical outcomes [30]. Classically activated, M1 macrophages usually activate Th1 cells to provoke cellular immunity and cytotoxicity and their presence within tumor islets have been associated with improved outcomes [31]. Alternatively activated M2 macrophages tend to function with Th2 cells, leading to tumor progression and immunosuppression[32]. In the tumor microenvironment the most frequently found immune cells are TAMs[33], which promote tumor growth, invasion and metastasis. To note, the phenotype associated with cancer initiation is considered to be the activated M1[8]. To the other extreme, in established tumors, the macrophage phenotype changes from the “inflammatory”, to the alternatively activated/trophic M2 phenotype of TAMs[34]. Interestingly, M2 polarized macrophages tend to release lower levels of IL-1β after NLRP3 activation[35]. In contrast to TAMs, AMs in lung cancer progression are not well studied. A study has provided evidence that AMs in lung tumor bearing mice, favor Th2 responses and facilitate metastasis[36]. Our research group has shown that lung specific involvement of the NLRP3 inflammasome is also impaired in idiopathic lung fibrosis, in agreement with the anti-inflammatory/pro-fibrotic profile of alveolar macrophages reported [22].

Inducible inflammatory responses in AMs from LC were decreased, as shown by our study. This could be either attributed to cancer-induced chronic inflammation resulting in immunosuppression, or it could describe a pre-existing condition resulting in the emergence of cancer. In the context of chronic inflammation, it has already been suggested that in COPD, a global impairment in TLR signaling and phagocytosis is evident[37]. Underlying lung disease (as pulmonary function testing revealed in terms of FEV1) was scarce and statistically insignificant. Thus, we propose that in the present cohort such an established independent variable of alveolar macrophage dysfunction (FEV1) could not be taken into account. Furthermore, impairment of AMs function has been previously associated with disease severity[38]. In our experiments, we used solely TLR4 stimulus, thus we cannot provide evidence of a global TLR signaling impairment in AMs.

Furthermore, it has been established that NLRP3 priming is an NF-kB dependent process[39] and chronic NF-kB signaling, leads to LPS tolerant macrophages[39]. Additionally, inhibition of NF-kB could reprogram TAMs towards an M1 phenotype [40]. Smoking can lead to NF-κB-induced chronic airway inflammation and accumulation of M2-polarized alveolar macrophages[41]. Smoking inhalation has been shown to increase serum TNF-α and IL-6 [42] and remain relatively undetectable in healthy non-smokers. Long-term exposure to tobacco smoke accounts for the majority of lung cancers. And individual chemical components of cigarette smoking, act as triggers for the inflammasome [43–45]. Furthermore ceramide, is highly induced due to cigarette smoking [46, 47], and is significantly increased in lung tissue from patients with smocking induced emphysema [46]. Of note ceramide increases oxidative stress and induces caspase-1 dependent and independent cellular events [48]. Due to the pleotropic interplay of smocking and the Inflammasome the observed variances could be attributed to smocking itself. However, patients enrolled in our study were smokers in both control and LC groups and thereafter we could conclude that the observed differences could mainly be attributed to the presence of lung cancer, rather than smoking.

It has been proposed that IL-1 mediates cytotoxicity and suppression of tumor growth [49, 50] and TNF-α inhibits angiogenesis to prevent tumor growth. In contrast, IL-1β has been shown to play a role in the development of the premetastatic niche[51] and targeting the inflammasome-IL-1β pathway been proposed to provide a novel approach for the treatment of cancer[52]. Previous studies have shown IL-1, IL-6, and TNF-α increase in lung cancer patients and these cytokines progressively decrease as the clinical stage of cancer progresses [11]. The ability of IL-6 to inhibit tumor cell growth has been suggested, as IL-6 functions synchronously with IL-1 and TNF-α in order to support anti-tumor immunity [53] and has been shown to regulate the ability of AMs in lung cancer to be stimulated by IFN-γ and LPS[54]. It was suggested that targeting IL-6 could potentially improve lung cancer therapeutic techniques [55]. Conversely, in a mouse model, chemoprevention of lung cancer was associated with reducing IL-6[56].

As previously suggested, decreased TNF-α and IL-1 secretion has been demonstrated in AMs from patients with both NSCLC and SCLC and reduced IL-6 secretion has been demonstrated from AMs derived from patients with large cell undifferentiated and small cell subtypes [54]. Importantly, a report in tumor-bearing mice, has suggested that AMs can produce higher levels of IL-1β, after LPS/ATP stimulation[57]. TNF-α, IL-1, and IL-6 promote the induction of Th1 cells, which enable macrophage-mediated killing. Reduced Th1 mediated cytokines may consequently limit the cytotoxic potential of AMs or TAMs and enable tumor progression [11]. Our results further add to the hypothesis that the deregulation of these cytokines may generate from a severely impaired NLRP3 pathway.

To note, caspase-1 inhibition, resulted in partial reduction of IL-1b and IL-18 secretion in all groups. Recent advances in the inflammasomes research field, have provided evidence that LPS alone could alternately activate NLRP3 to secrete IL-1b. In our experiments we treated cells with caspase-1 the inhibitor following two hours LPS treatment, and the remaining IL-1β could be attributed to that effect of LPS in all groups. Furthermore, IL-1β was virtually undetected in the cytoplasm of any of the LC samples, while we hypothesize that cytoplasmic retention of IL-1β in controls may represents the collection time, rather than secretion being hindered.

The role of high IL-18 secretion by lung cancer AMs and PBMCs as observed here, in carcinogenesis remains unclear, since it exerts both antitumor and protumor effects. IL-18 has the ability to inhibit the recognition of cancer cells by immune cells, increase cancer cell adherence to the microvascular wall, induce the production of angiogenic and growth factors, and promote a pre metastatic microenvironment[58]. We also noted a differential release of IL-1β and IL-18 at baseline from AMs. Evidence suggests that the expression of inactive pro-IL-1β and pro-IL-18 is differentially regulated by different stimuli [59] and our results at steady state, prior to activation could represent a pre-exposure to a certain stimulus.

By contrast to the lung microenvironment, systemically we observed a typical pro-inflammatory pattern in LC. LC PBMCs produced high levels of IL-1β, IL-18, and IL-6, whereas low levels of TNF-α, and could be furthermore stimulated in the presence of LPS. In accordance with our findings, a recent research reported that IL-1β is elevated in the serum of patients with NSCLC and further *in vitro* studies suggested that IL-1β promoted the proliferation and migration of NSCLC cells[60]. Increased levels of IL-1 and IL-6 are noticed in lung cancer patients and progressively decrease as cancer progresses [11]. Of note, exploratory data from a large randomized trial has provided evidence that in subjects with high CRP, yet otherwise healthy, inhibition of IL-1β by canakinumab, reduced the incidence of lung cancer, as well as the lung cancer related mortality. These discrepancies in the periphery, observed by our study, could represent a pro-inflammatory state of individuals leading to the development of Lung cancer. By contrast, in the lung microenviroment the decreased TLR4/NLRP3 axis could represent a tumor mediated immunosuppression.

In our experiments, although unstimulated PBMCs from LC patients surpassed controls in IL-6 levels, stimulation of the TLR4 pathway did not induce IL-6 secretion in either group. In contrast, TNF-α secretion by unstimulated PBMCs was lower in lung cancer than controls, while maintaining inducibility status via the TLR4/LPS pathway. TNF-α is a pro-inflammatory cytokine that is cytotoxic for tumor cells. It has been demonstrated that increased TNF-α levels in NSCLC correlate with improved outcomes, while TNF mutants have been proposed as anticancer chemotherapeutic drugs [61, 62]. This dichotomous result in terms of antitumor immunity profiling could suggest a deregulation of the innate immunity mechanisms early on in the inflammasome process.

Our study does not lack limitations. A direct comparison between AMs in lung microenvironment and PBMCs was not the scope of our study, but we rather aimed to characterize the inflammasome activity in the two distinct compartments (tumour lung environment and the periphery). Peripheral–Blood monocytes and macrophages have well established differences in TLR signaling and immune cell trafficking with AMs exist in both healthy [63, 64] and COPD patients [65, 66], which comes in agreement with our findings. Another limitation of our study is that, only TLR4 stimulation by LPS was used and a global TLR impairment in the LC AMs cannot be excluded, as already reported in COPD AMs[37] [38]. Additionally, COPD often underlies LC and as a disease is highly linked to macrophage dysfunction[37], hence the involvement of patients with COPD-comorbidity in our study is a further limitation that should be acknowledged. Apart from the COPD enrollment, the LC group was consisted from LC patients irrespective of the different histological subtypes and thereafter is very heterogeneous. However, from our analysis no differences arose in any of the studied cytokines based on histological subtype.

## Conclusion

Through cytokine phenotyping, we suggest that alveolar macrophages, in lung cancer microenvironment, are impaired. In AMs the activation of TLR4/NLRP3 inflammasome was found severely decreased, adding to the hypothesis of cancer mediated immunesuppression. By contrast, systemically a pro-inflammatory state was observed, which may constitute a pro-inflammatory environment that leads to cancer incidence and progression. To date, several antagonists have being developed against components of the inflammasome, and have been proposed as anti-cancer therapy. Novel targeting of IL-1β could reduce the incidence of Lung cancer in healthy individuals, as already suggested [18], however, such treatment should be used with caution in established lung cancer, since our results suggest that NLRP3 inflammasome pathway is already impaired in lung cancer microenvironment. Given the ability of NLRP3 in promoting immunogenic cell death, the activation/ reprogramming of dysfunctional AMs, could be a novel add-on therapy in established lung cancer.

## Supporting information

S1 Fig Outline of inflammasome stimulation conditions. (TIF)

S2 Fig Intracellular levels of IL-1b. (TIF)
