# Supplementary figures and images for: NLRP3/Caspase-1 inflammasome activation is decreased in alveolar macrophages in patients with lung cancer
Source: PLoS One. 2018 Oct 26;13(10):e0205242. doi: 10.1371/journal.pone.0205242 (PMC6203254; doi:10.1371/journal.pone.0205242)

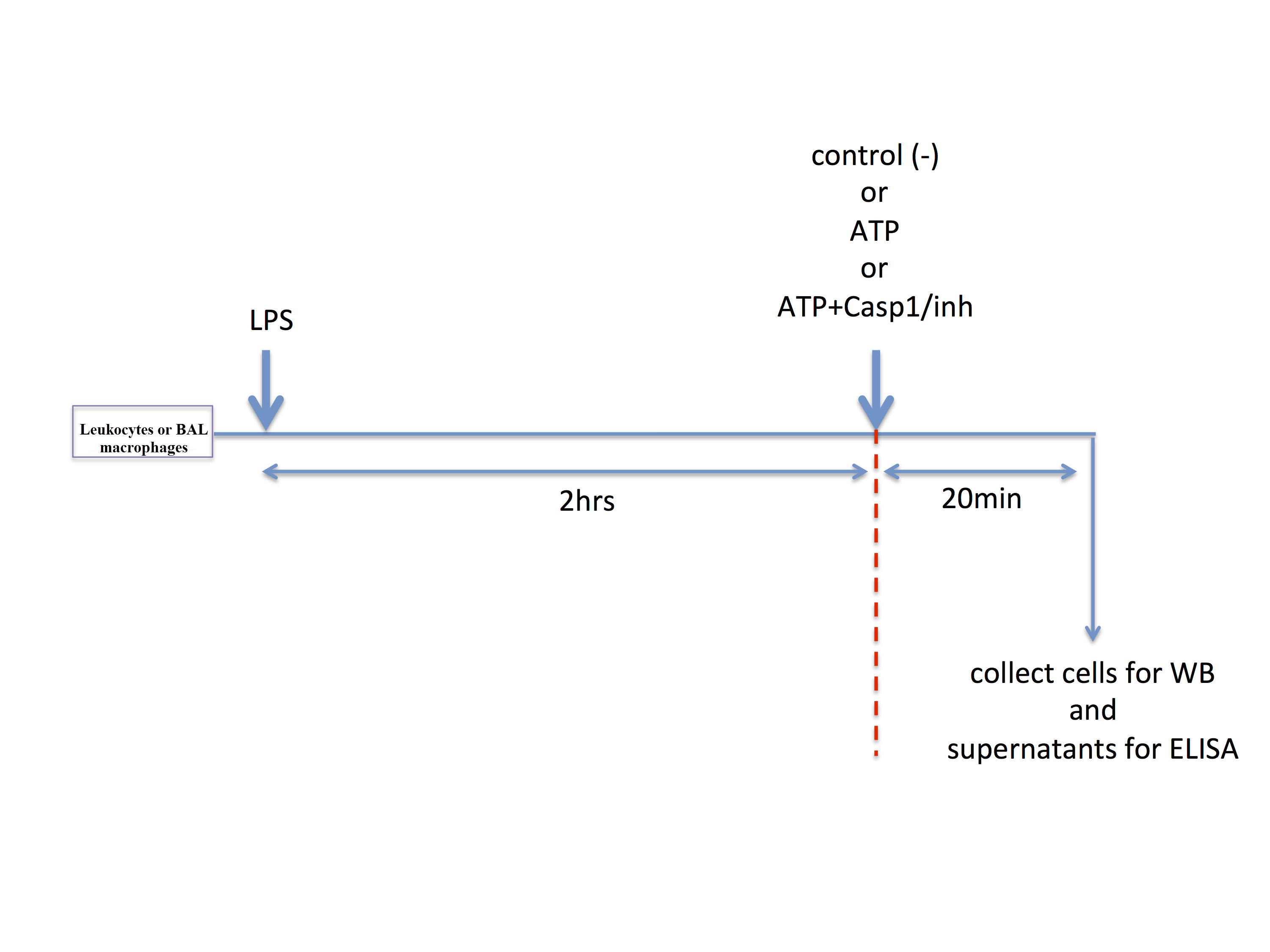

Supplement: S1 Fig — (TIF) [file pone.0205242.s001.tif]

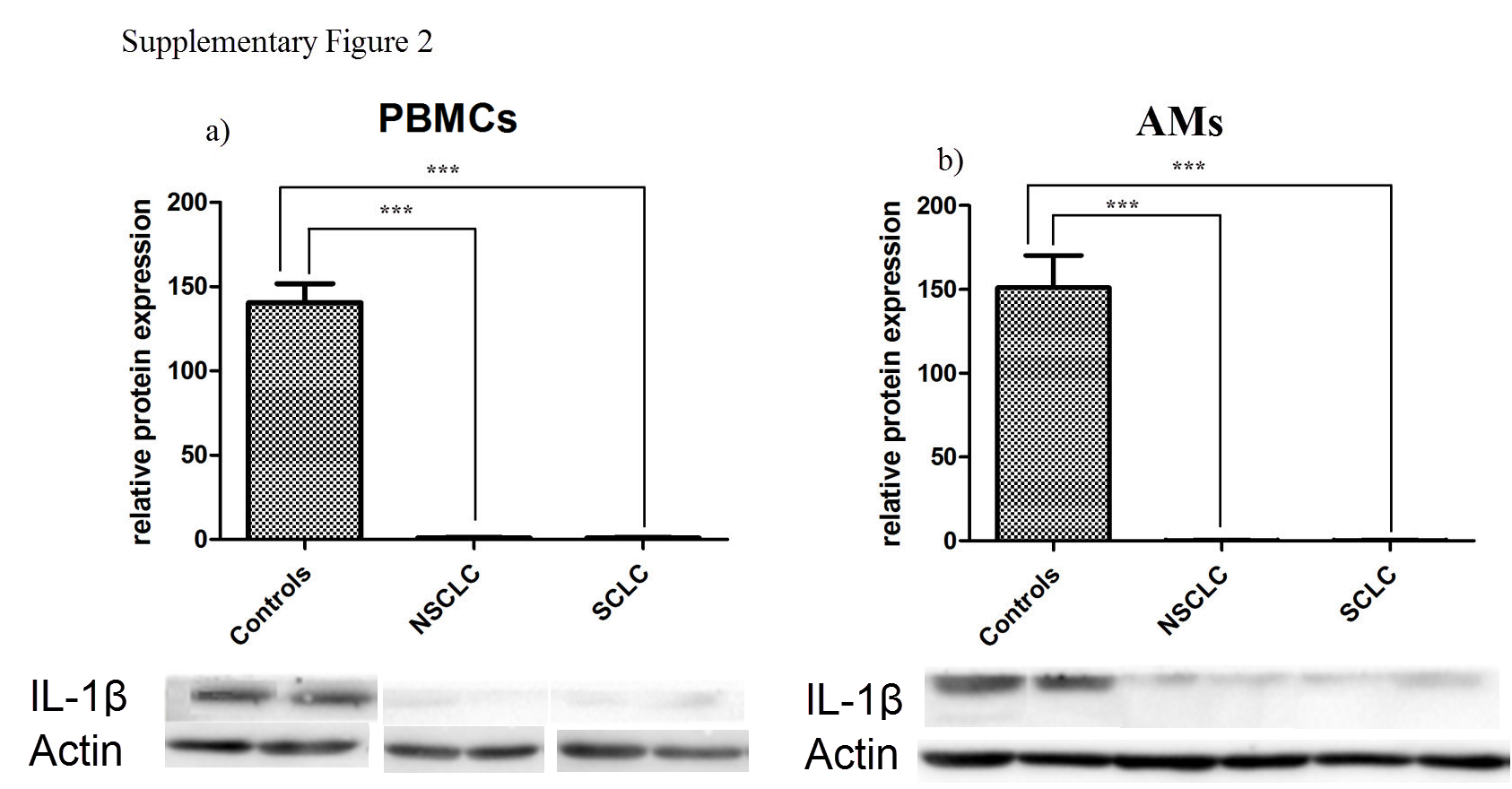

Supplement: S2 Fig — (TIF) [file pone.0205242.s002.tif]
